# Cohort Profile: The PERU MIGRANT Study–A prospective cohort study of rural dwellers, urban dwellers and rural-to-urban migrants in Peru

**DOI:** 10.1093/ije/dyx116

**Published:** 2017-07-25

**Authors:** Rodrigo M Carrillo-Larco, Andrea Ruiz-Alejos, Antonio Bernabé-Ortiz, Robert H Gilman, Liam Smeeth, J Jaime Miranda

**Affiliations:** dyx116-1 CRONICAS Center of Excellence in Chronic Diseases, Universidad Peruana Cayetano Heredia, Lima, Peru,; dyx116-2 Faculty of Epidemiology and Population Health, London School of Hygiene and Tropical Medicine, London, UK,; dyx116-3 Department of International Health, Bloomberg School of Public Health, Johns Hopkins University, Baltimore, MD, USA and; dyx116-4 Department of Medicine, School of Medicine, Universidad Peruana Cayetano Heredia, Lima, Peru

## Why was the cohort set up?

Infectious diseases are still a major concern in developing countries, causing up to 60% of the deaths in low-income countries. However, non-communicable diseases (NCDs) and their associated risk factors are becoming a major public health issue in the developing world where between 38% (low-income) and 80% (upper middle-income) of deaths are attributable to NCDs.^1^^,^^2^ Major common risk factors include raised blood pressure, elevated blood glucose, obesity, low physical activity, unhealthy diet habits, smoking and alcohol consumption. Many risks are associated with lifestyle and have rapidly changed over recent decades, some driven by urbanization.^3^^,^^4^

Environmental changes as well as population flow have led to an increase in urbanization. The degree of urbanization is closely linked to distinctive features of the health profile of rural and urban participants, as well as of rural-to-urban migrants. Internal migration led to an ‘urbanization’ of the individuals who were born in rural settings and causes a change in their lifestyle habits. Low physical activity, for example, is linked to urban settings whereas agriculture, the main labour in the rural area, implies high physical activity.

Within-country migration is a complex phenomenon and can be driven by many reasons, including but not limited to the aspiration to seek better socioeconomic standards or living conditions, war displacement and natural disasters. The PERU MIGRANT Study (PEru's Rural to Urban MIGRANTs Study) was conducted to identify the impact of rural-to-urban migration on selected cardiometabolic outcomes, e.g. obesity, type-2 diabetes and hypertension. The study includes rural-to-urban migrants as well as rural and urban non-migrant groups.^5^ Because of political violence between the 1970s and 1990s, approximately 120 000 families moved from rural highlands to urban coastal settlements in Peru.^6^^,^^7^ This scenario offered an opportunity to study rural-to-urban migration without the potential bias introduced by socioeconomic improvement, thus decreasing the impact of selection bias.

The research questions addressed by the PERU MIGRANT Study were:

- Is there a difference in specific cardiovascular disease (CVD) risk factors in rural-to-urban migrants compared with those rural or urban dwellers who did not migrate?
- Do CVD risk factor patterns among migrant populations vary by:
  - length of residence in urban environment?
  - lifetime exposure to urban environment?
  - age at first migration?

We considered analysing the exposure to the urban environment as years and percentage of life exposure. Thus, the length of urban residence was considered as the number of years that the rural-to-urban migrants reported to had lived in an urban setting. On the other hand, lifetime exposure was the percentage obtained by dividing the number of years lived in an urban area by participant’s current age in years.

### Settings

The PERU MIGRANT Study was conducted in Lima, an urban sea-level setting, and Ayacucho, a rural high-altitude setting located at 2761 m above sea level. The reason for choosing these sites was for convenience. The research team had conducted other studies previously in these settings, or were close to researchers who had worked there previously. Ayacucho is an Andean department considered one of the areas most affected by political violence that occurred between 1970 and 1980. About 50% of all deaths caused by terrorism occurred in this area.^7^ Approximately 11% of the total migrants to Lima were from Ayacucho.^8^ These figures made Ayacucho the leading source of rural-to-urban migrants to Lima. We selected the village of San Jose de Secce, in the district of Santillana, province of Huanta, in Ayacucho as the rural study site. In San Jose, 50% of the population is considered extremely poor, with only 5% of residents having direct access to potable water. In addition, the literacy rate is around 60% and the main language is Quechua.^9^ The peri-urban shanty town called Las Pampas de San Juan de Miraflores, in the south of Lima, was chosen as the urban study site. The definition we consider for ‘urban’ is having approximately 100 houses clustered. The population in this area is ‘extremely poor’ and up to 20% is ‘poor’. Literacy rate is 79% and the main language is Spanish. Both sites represent the urban, migrant and rural populations due to the number of participants and environmental characteristics. Funding for the baseline assessment was provided by the Wellcome Trust. The first follow-up assessment was partly funded by the Universidad Peruana Cayetano Heredia. The second follow-up round was partly funded by the GloCal Health Fellowship Program from the University of California Global Health Institute.

## Who is in the cohort?

In 2007, adult subjects from San Jose de Secce rural group were identified after a census was made. The 2006 updated Las Pampas de San Juana de Miraflores census was used to identify urban residents who were born in Lima (urban dwellers) and who were born in Ayacucho (rural-to-urban migrants). Participants were recruited using a single-stage random sampling technique that was sex- and age-stratified (30–39, 40–49, 50–59 and ≥60 years) using name, address and national identification number. Men and women ≥ 30 years old and permanent residents were considered eligible for the study. Pregnant women and those with mental conditions that would have prevented completion of the study procedures were excluded.

Power calculations were derived using conservative estimates of the prevalence of major risk factors in the areas of Huaraz (urban, Andes) and Ingenieria (urban, Lima). The baseline survey, conducted in 2007–08, was designed to include 1000 participants: 200 born in Ayacucho who have always lived in rural areas, 600 rural-to-urban migrants born in Ayacucho and living in Pampas de San Juan de Miraflores in Lima and 200 urban participants who have always lived in urban areas. Comparing the Lima with the Andes group, at least 200 people in each group would give a power of 80% and a significance level of 5% to detect a difference in the prevalence of hypertension (33% vs 19.5%), hypercholesterolaemia (22.7% vs 10.6%) and diabetes (7.6% vs 1.3%). More rural-to-urban individuals were included to have further information from this group, because additionally, this group was expected to be divided into two groups to be analysed according to migration surrogates.

A total of 1606 dwellers were invited to participate in the study. The general response rate at enrolment was 73.2% (1176/1606) and the overall response rate at completion was 61.6% (989/1606). Response rate was the highest in the rural group (84.8%), and the corresponding figures were 56.8% and 77.7% for urban and migrant groups, respectively. Further details about sample size and sample enrolment at baseline are available elsewhere.^5^ Characteristics of the individuals who refused to participate in the baseline assessment, and reasons for refusal, have been previously published,^5^ including having access to health care and thus not needing the health evaluation provided by the study, and logistical issues e.g. time constraints due to work or travel.^5^

Before participation, an informed consent was signed by the participant. The protocol of the study was approved by the institutional review board of the Universidad Peruana Cayetano Heredia.

## How often have they been followed up?

**Figure 1:**
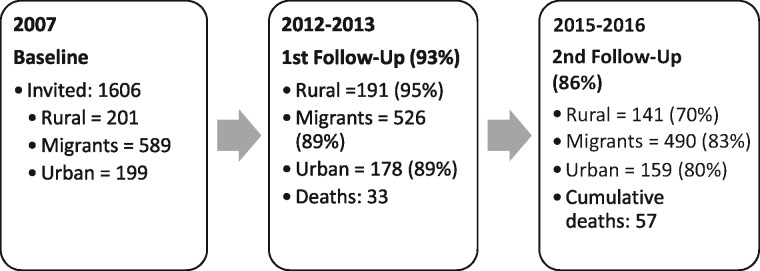
Number of participants in each round of the PERU MIGRANT Study. The percentages for follow-up rates do not include deaths in each group.

Five years after the baseline assessment, in 2012–13, the participants of the PERU MIGRANT Study were re-contacted for the first follow-up. A second follow-up assessment was completed in June 2016. The response rates for the first and second follow-up rounds were 93.8% and 85.6%, respectively (Figure 1 ).

The first follow-up evaluation aimed to revisit all 989 participants recruited at baseline. In this round, 33 deaths were recorded and 61 participants were lost to follow-up (Figure 1). Sociodemographic characteristics of participants re-contacted or lost to follow-up at the first follow-up round are presented in Table 1 and Table 2.

**Table 1:** Characteristics of PERU MIGRANT Study participants at first follow-up round: deaths, lost-to-follow up and re-contacted

	Re-contacted participants		Lost-to- follow-up participants
	Dead *N* = 33	Alive *N* = 895	Overall *N* = 61
Migration status			
Rural	10 (30.3)	191 (21.3)	0 (0.0)
Migrant	14 (42.4)	526 (58.8)	49 (80.1)
Urban	9 (27.3)	178 (19.9)	12 (19.9)
Age (years)			
30–39	3 (9.1)	259 (28.9)	20 (32.8)
40–49	1 (3.0)	263 (29.4)	18 (29.6)
50–59	7 (21.2)	252 (28.2)	13 (21.4)
60+	22 (66.7)	121 (13.5)	10 (16.4)
Sex			
Male	22 (66.7)	413 (46.1)	32 (52.5)
Female	11 (33.3)	482 (53.9)	29 (47.5)
Education			
Primary or less	24 (72.7)	430 (48.4)	26 (42.6)
Some secondary or more	9 (27.3)	463 (51.6)	35 (57.4)
Asset index			
Lowest	19 (57.8)	313 (35.0)	15 (24.6)
Middle	8 (24.2)	294 (32.9)	25 (40.9)
Highest	6 (18.1)	288 (32.2)	21 (34.4)

The entries in parentheses refer to the corresponding percentages (%).

**Table 2:** Characteristics of PERU MIGRANT Study participants at first follow-up round, i.e. deaths, lost-to-follow-up and re-contacted according to study group

	Re-contacted participants			Lost-to-follow-up participants
	Rural *N* = 201	Migrant *N* = 540	Urban *N* = 185	Overall *N* = 61
Mortality	10 (5.0)	14 (2.6)	9 (4.8)	–
Age (years)				
30–39	20 (9.9)	64 (11.9)	23 (12.4)	60 (98.4)
40–49	59 (29.4)	157 (29.4)	48 (25.9)	1 (1.6)
50–59	43 (21.4)	158 (29.3)	57 (30.8)	–
60+	55 (27.4)	135 (25.0)	47 (25.4)	–
Sex				
Male	95 (47.3)	252 (46.7)	88 (47.1)	32 (52.5)
Female	106 (52.7)	288 (53.3)	99 (52.9)	29 (47.5)
Education				
None/some	132 (65.6)	168 (31.2)	13 (6.9)	15 (24.6)
Primary complete	30 (14.9)	92 (17.1)	19 (10.2)	11 (18.0)
Some secondary or more	39 (19.4)	279 (51.7)	154 (82.8)	35 (57.4)
Asset index				
Lowest	196 (97.5)	108 (20.0)	28 (14.9)	15 (24.6)
Middle	5 (2.5)	233 (43.2)	64 (34.2)	25 (40.9)
Highest	0 (0.0)	199 (36.9)	95 (50.8)	21 (34.4)

The entries in parentheses refer to the corresponding percentages (%).

Updated figures for 2016, after the second follow-up visit, include 57 deaths recorded and 142 participants lost to follow-up. Accordingly, to date, information from 847 participants, divided into 154 rural dwellers, 520 rural-to-urban migrants and 173 urban individuals, is available. The cumulative mortality after the second follow-up was 6.7% using 989 as the population’s denominator. Characteristics of re-contacted participants at the second follow-up round are presented in Table 3.

**Table 3:** Characteristics of PERU MIGRANT study participants at second follow-up

	Re-contacted participants		
	Rural *N* = 154	Migrant *N* = 520	Urban *N* = 173
Mortality	13 (8.4)	30 (5.8)	14 (8.1)
Age (years)			
30–39	–	–	–
40–49	41 (29.5)	147 (30.3)	43 (26.7)
50–59	37 (30.3)	153 (31.5)	53 (32.9)
60+	61 (26.7)	186 (38.7)	65 (40.4)
Sex			
Male	63 (40.9)	241 (46.4)	77 (44.5)
Female	91 (59.1)	279 (53.7)	96 (55.5)
Education			
None/some	101 (65.6)	164 (31.6)	12 (7.0)
Primary complete	22 (14.3)	90 (17.3)	18 (10.4)
Some secondary or more	31 (20.1)	265 (51.1)	142 (82.6)
Asset index			
Lowest	149 (96.8)	103 (19.8)	29 (16.8)
Middle	5 (3.5)	222 (42.7)	60 (34.7)
Highest	0 (0.0)	195 (34.7)	95 (48.6)

The entries in parentheses refer to the corresponding percentages (%).

## What has been measured?

The baseline assessment of the PERU MIGRANT Study aimed to identify prevalence of CVD risk factors and major NCDs. These included: obesity, defined as BMI ≥ 30; hypertension, considered as the mean of three blood pressure measurements ≥ 140/90, or previous physician diagnosis or currently receiving treatment for hypertension; type 2 diabetes mellitus, considered in those with fasting glucose ≥ 126 mg/dl, or previous physician diagnosis or currently receiving treatment for diabetes. Dyslipidaemia was considered as total cholesterol ≥ 200 mg/dl, triglycerides ≥ 200 mg/dl, low-density lipoprotein (LDL) ≥ 160 mg/dl and high-density lipoprotein (HDL) ≤ 40 mg/dl for men and ≤ 50 mg/dl for women. Cardiovascular diseases (myocar-dial infarction and stroke) were considered as the self-report of previous diagnosis by a physician. Other risk factors assessed were: physical activity and tobacco and alcohol consumption.

The follow-up rounds were conducted to study the incidence and risk of those NCDs and their associated risk factors. In addition, in order to better determine CVD, the second follow-up included an electrocardiographic evaluation in which signs of necrosis were considered for diagnosis. Variables collected throughout study rounds are summarized in Table 4. In brief, information was collected using: face-to-face questionnaires including sociodemographic variables, lifestyle behaviuors and self-reported clinical conditions; the clinical evaluation, e.g. anthropometric procedures and electrocardiogram; and blood samples, e.g. lipid profile, fasting glucose and inflammatory markers, among others.

**Table 4:** Information collected at each study round, the PERU MIGRANT Study

Phase	Information collected	
Baseline 2007–08	Laboratory	Fasting glucose, fasting insulin, glycosylated haemoglobin, lipid profile (total cholesterol, HDL-c, triglycerides), high ultrasensitive C-reactive protein, self-reported socioeconomic position
	Clinical examination	Anthropometric measurements: weight, waist and hip circumferences, skinfolds (subcapsular, biceps, triceps and supra-iliac), blood pressures
	Questionnaire	Self-reported NCDs (myocardial infarction, stroke, type 2 diabetes mellitus, dyslipidaemia), alcohol consumption, smoking status, mental health (12-item general health questionnaire), medication (specially antihypertensive and hypoglycaemic agents), socioeconomic factors, physical activity level (IPAQ), a social capital instrument validated in Peru, acculturation scale, Rose angina questionnaire
First follow-up 2012–13	Clinical examination	Anthropometric measurements: weight, height, waist and hip circumferences, blood pressures
	Questionnaire	Self-reported NCDs, alcohol consumption, smoking status, mental health (12-item general health questionnaire), medication (specially antihypertensive and hypoglycaemic agents), verbal autopsy and death certificates if available
Second follow-up 2015–16	Laboratory	Fasting glucose
	Clinical examination	Weight, body composition (bioimpedance) and blood pressure measurements, assessment using electrocardiogram (ECG)
	Questionnaire	Questions related to the occurrence of cardiovascular diseases and/or risk factors such as previous diagnosis of myocardial infarction, hypertension, stroke, diabetes, physical activity level (IPAQ); plus self-rated status of health, medication and the patient health questionnaire (PHQ-9; mental health status)

Personal histories of cardiovascular diseases and other non-communicable diseases were self-reported.

## What has it found? Key findings and publications

Major findings from the baseline assessment identified a clear pattern of differences in cardiovascular risk factors according to migration status.^10^ The length of urban residence had a robust impact on the prevalence of obesity in rural-to-urban migrants: 12% higher obesity prevalence was observed for each additional 10-year period of urban residence [95% confidence interval (CI) 6‐18].^11^ At baseline, prevalences of overweight, obesity and low physical activity were higher in the urban and migrant groups, relative to the rural group (*P* for trend = 0.001).^12^^,^^13^ Predictably, urban participants were almost 33 times more likely to have low physical activity [odds ratio (OR) 32.98; 95% CI 11.02‐98.63].

Hypertension prevalence was higher in the urban (29%) and migrant (16%) groups; however; the difference in prevalence between the migrant and rural groups (11%) was not significant.^10^ On the other hand, the overall prevalence of diabetes was 4.5% with a significant difference between groups (0.8%, 2.8% and 6.3% for rural, migrant and urban groups, respectively, *P* < 0.01).^14^ Higher odds of impaired fasting glucose, metabolic syndrome and diabetes were found in participants who migrated at age ≥ 12 years vs their peers who migrated at younger ages.^14^ A suboptimal control rate of hypertension was found in 95% of the hypertensive participants and 100% of those with diabetes, considering controlled those with blood pressure and HbA1c normal levels. For either or both conditions, treatment rates were higher in the urban than the migrant and rural groups, with a total of only 40% currently on medication.^14^

Data from the first follow-up round addressed four main issues: all-cause and specific-cause mortality;^15^ hypertension incidence;^16^ obesity incidence;^17^ and low HDL-cholesterol as a cardiovascular risk factor.^18^ In both follow-up rounds, mortality data were collected through verbal autopsy and death certificates when available. In addition, in the second follow-up, since we could not re-contact all the participants from the baseline, we requested information from the national death records.

Of the 33 deaths recorded in the first follow-up evaluation, nine were due to CVDs and eight due to cancer of unknown aetiology. Other causes included sepsis, accidental injuries and asthma, among others. In six cases, cause of death was undetermined. Men, older participants and individuals with hypertension, as well as those with lower education levels or a low assets index, were more likely to have died. There was a trend towards lower CVD mortality in migrant and rural dwellers, relative to urban counterparts. As such, urban dwellers were at higher risk of all-cause mortality compared with rural dwellers.^15^

Regarding hypertension, the rural group showed greater risk of developing hypertension, when compared with their urban counterparts, and central obesity explained most of the new hypertension cases observed across study groups.^16^ Relative to rural dwellers, the urban and migrant groups showed greater incidence of obesity. Migrant and urban participants showed an 8- and 9.5-fold higher incidence ratio of obesity compared with the rural group, respectively. Central obesity was the highest in the migrant group and its incidence ratio was associated with a higher assets index.^17^ Finally, individuals with non-isolated low HDL-cholesterol had a 2- to 3-fold higher risk of CVD, including fatal stroke and myocardial infarction, at the first follow-up assessment. Furthermore, lower levels of HDL-cholesterol were found in the rural group compared with their migrant and urban counterparts.^18^

## What are the main strengths and weaknesses?

The PERU MIGRANT Study followed three well-defined population groups: rural dwellers, rural-to-urban migrants and urban participants. The strength of the PERU MIGRANT Study does rely in its well-defined population groups: rural, urban and rural-to-urban migrants. A frequent and potential limitation of migration studies rests in the self-selection of the migrant participants due to better socioeconomic standards. Therefore, a strength of this cohort is that the migrants moved to urban settings due to political violence events, reducing the risk of socioeconomic selection bias. Finally, we re-contacted most of the initial study sample, particularly those in rural settings. Having completed two extensive follow-ups over an 8-year period, this cohort of rural-to-urban migrants and non-migrants is an asset for studies arising from low-income settings.

Still, the PERU MIGRANT Study has several limitations. First, the sample size is rather small and statistical power for some analyses is restricted. Second, most of the refusals at baseline were observed in the oldest old and in male participants; however, the final cohort studied included similar proportions of sex and age, minimizing the selection bias. Furthermore, urban individuals who rejected participation in the study had higher education levels, compared with those enrolled in that group. This fact could be associated with a low socioeconomic status and less access to health care adding selection bias.^5^ Finally, neither at baseline nor at the first follow-up round did we collect information about dietary patterns. Nevertheless, at the second follow-up a questionnaire to assess fat intake (e.g. low or high) was included.^19^ This caveat could be overcome with the use of assumptions in interpreting results. For example, relative to rural participants, their urban fellows would consume more fat/energy-dense foods, and so would migrants.^20^

In the future, further funding would enable a full assessment of markers in blood samples to complement the panel obtained at baseline, as well as measurements of fasting glucose obtained during the second follow-up, allowing for more comprehensive time variation analysis of glucose, lipid profiles, HbA1C and inflammatory markers.

## Can I get hold of the data? Where can I find out more?

Baseline data can be freely accessed online at [https://figshare.com/articles/PERU_MIGRANT_Study_Baseline_dataset/3125005]. If interested in establishing collaborations, conducting analyses or having further information regarding the PERU MIGRANT Study, please send an e-mail to our research centre at [cronicas@oficinas-upch.pe]. Should you wish to use our data, please contact us with a brief analysis plan: title, research question, general or specific objectives, main variables and statistical analysis plan. Further information about our research group can be found at [http://en.cronicas-upch.pe/].

## Funding

The establishment of the PERU MIGRANT Study was funded through a Wellcome Trust Master Research Training Fellowship and a Wellcome Trust PhD Studentship to J.J.M. (074833/Z/04/A). The first follow-up evaluation was funded by Universidad Peruana Cayetano Heredia (Fondo Concursable No. 20205071009). The second follow-up evaluation was funded by the National Heart, Lung, and Blood Institute, National Institutes of Health, through the GloCal Health Fellowship Program from the University of California Global Health Institute. R.M.C-L., A.B-O., J.J.M. and the CRONICAS Centre of Excellence in Chronic Diseases were supported by Federal Funds from the United States National Heart, Lung, and Blood Institute, National Institutes of Health, Department of Health and Human Services, under contract No. HHSN268200900033C. L.S. is a Wellcome Trust Senior Clinical Fellow (098504/Z/12/Z), and A.B-O. is a Wellcome Trust Research Training Fellow in Public Health and Tropical Medicine (103994/Z/14/Z).

**Conflict of interest:** The authors declare no conflict of interest.

**PERU MIGRANT Study profile in a nutshell**

- The cohort was established to study cardiovascular diseases and associated risk factors in three population groups in Peru: rural, urban and rural-to-urban migrants. The PERU MIGRANT Study’s posed hypothesis was that the occurrence and progression of cardiovascular disease and their risk factors would be different among these groups.
- Peru offers an unusual scenario to study rural-to-urban migration: a lot of migration happened in response to violence rather than economic issues, with a reduced likelihood of selection effects.
- The study was conducted in Ayacucho, a rural site, 2761 m above sea level, and in Lima, an urban site at sea level. The baseline assessment was in 2007–08, with 989 participants aged ≥ 30 years.
- There have been two follow-up evaluations conducted in 2012–13 and 2015–16. Out of the 989 people enrolled at baseline, 57 deaths were recorded and 142 participants were lost to follow-up, considering both study rounds. Although the second follow-up has been completed, the PERU MIGRANT is an ongoing cohort. We expect to re-contact the participants in the near future.
- Information collected throughout baseline, first and second follow-up rounds includes participants’ sociodemographic, behavioural and medical history and clinical data. Blood samples were taken at baseline and at the second follow-up evaluation only.
- Baseline data are available online at [https://figshare.com/articles/PERU_MIGRANT_Study_Baseline_dataset/3125005]. Collaboration proposals or requests for further information should be e-mailed to [cronicas@oficinas-upch-pe].
